# Mentalization and dissociation after adverse childhood experiences

**DOI:** 10.1038/s41598-022-10787-8

**Published:** 2022-04-26

**Authors:** J. Wagner-Skacel, D. Riedl, H. Kampling, A. Lampe

**Affiliations:** 1 Department of Medical Psychology and Psychotherapy, Medical University Graz, Graz, Austria; 2 Department of Psychiatry, Psychotherapy, Psychosomatics and Medical Psychology, Medical University Innsbruck, Innsbruck, Austria; 3 Department of Psychosomatic and Psychotherapy, Justus Liebig University Giessen, Giessen, Germany; 4 VAMED Rehabilitation Center, Schruns, Austria; 5 Ludwig Boltzmann Institute for Rehabilitation Research, Vienna, Austria

**Keywords:** Psychology, Human behaviour, Medical research

## Abstract

.

Impairment of mentalization may impact coping strategies, regulation of affect and stress. The influence of impaired mentalization on dissociation in patients with adverse childhood experiences (ACEs) could be important for treatment strategies. The aim of this study is to assess the relationship between ACEs, mentalizing and dissociation in adult individuals. Sixty-seven patients with ACEs completed the Mentalization Questionnaire (MZQ), the Essener Trauma Inventory (ETI) and the Brief Symptom Inventory-18 (BSI-18). The SPSS PROCESS macro tool was applied to test if mentalization mediated the relationship of ACEs and dissociation. ACEs were significantly associated with higher dissociation (β = 0.42, p < 0.001) and lower mentalization (β = − 0.49, p < 0.001). When mentalization was added to the model as a predictor, the association of ACEs with dissociation was no longer significant (β = 0.11, p = 0.31) and a statistically significant indirect effect was found (β = 0.32, 95% CI 0.16–0.47). The overall explained variance of dissociation notably improved after inclusion of mentalization (17.5% to 49.1%). Thus, the results indicated that the association of ACEs on dissociation was fully mediated by mentalization. Our results suggest that ACEs are associated with lower mentalization and higher dissociation. Lower mentalization was also associated with worse depression, anxiety, somatization and PTSD symptoms. These findings underline the increasing importance of early treatment of individuals affected by ACEs with a focus to foster the development of mentalization.

## Introduction

Dissociation is a mental process which allows an individual to tolerate distressed events by splitting off highly incoherent or overwhelming thoughts, memories and feelings^1^. A disruption is a disconnection in the integration of consciousness, memory, identity, emotion, perception, body representation, motor control and behavior. It affects all areas of personality functioning and of the integration of self^2^. The dissociative process could be understood as a primary response to stress related with morphological alterations in the brain and is linked with enhanced amygdala response to emotional cues and cognitive control^2,3^. There is considerable evidence regarding the relationship between childhood abuse or neglect and dissociation symptoms in adulthood^4^ with in an earlier age of onset, as well as a longer duration of abuse and parental abuse significantly predicted higher dissociation scores^5^. Chronic exposure to a stressful environment may lead to several alterations^5^. In line with psychodynamic theory, experiences of childhood trauma play a crucial role in the etiology of psychiatric disorders and hence show somatic diseases epigenetic modifications of the glucocorticoid receptor gene on the stress response in several studies with individuals with childhood abuse^6^. Childhood abuse is regarded as an event so intense that it is impossible for the victim to integrate this experience on a symbolic level and thus fosters a pathological active formation of impaired personality functioning and affective forces^6^.

Thus, it is reasonable that such adverse childhood experiences (ACEs) might hinder the development of good mentalizing abilities. Mentalizing is defined as the process by which we make sense of each other and ourselves with forming beliefs about mental states of those with whom we interact and our own^7^.

Mentalization can be seen as an extension and complement to the theory of mind research and to other approaches of perspective shifting competency with a special consideration of the processing of biographically relevant relationship experiences. The clinical concept particularly focuses on the area of metacognitive access to one's own self-states, attachment representations and interactions in affective contexts^8^. Mentalizing as a key aspect of social cognition is a highly complex ability based on two types of explicit and implicit mentalization^9^. Implicit is characterized by fast and intuitive, pre-reflexive and non-verbal information process without awareness. The explicit form of mentalizing is relatively slow and relies on verbal and conscious information processing with cognitive skills^9^. In many previous studies the concept has been analyzed in relation to childhood trauma and dissociation. A significant interaction can be seen between adverse childhood experiences and attachment security with more post-traumatic stress disorder and trauma symptoms in those with insecure attachment representation^10^. Attachment style may play a role in precipitating and protecting against post-traumatic stress^11^. Trauma is theorized to activate the attachment system, so there is a reciprocal relationship between attachment and trauma.

Mentalization is a dynamic multifaceted ability that has particular salience in the context of attachment relationship^12^. Several studies documented trauma-induced neurobiological changes within the amygdala^13^, recognized as a primary locus of differentiated reactions to trauma and long-term socio-emotional consequences^14^. Neuroimaging studies show amygdala hyperactivity in individuals with acute trauma exposure heightened emotional response (e.g. hyperarousal, re-experiencing) and blunted emotional response (e.g. emotional numbing, dissociation) in cases of early and prolonged onset^14^.These blunted emotional responsiveness has been seen in mothers with unresolved trauma to infant distress^15^. Attachment trauma results in an increased amygdala response to salient stimuli^16^. For the child who is exposed to violence and abuse, there is hardly any opportunity to reflect on their own inner world as they are forced to concentrate exclusively on the external traumatic world^17^.

Emotional processes are linked to cognitive operations and reflective awareness with a language based broad spectrum of complex memory. Cognitive and executive functions are linked to mentalization identity narratives and mindfulness^18^. The relation of cognition and emotion with the underlying structure of personality might be important when considering personality functioning and vulnerability. Personality changes can lead to distinct impairments in self and interpersonal function^19^. Internalized early adverse experiences lead to corresponding inner working models that obstruct the functional regulation of dissociation. These mental processes might be modified in stressful circumstances and aggravate to difficulties.

**Figure 1:**
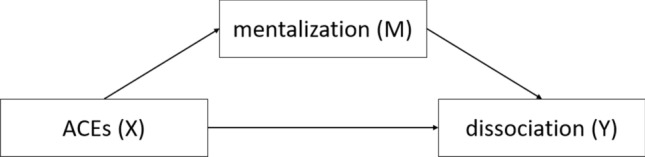
Theoretical model of the moderating effect of mentalization on the relationship of ACEs and dissociation.

It is reasonable to assume that the psychophysiological processes of mentalization may be involved in the dynamic of dissociation with its impact on imagination, our various verbal and conceptual information, as well as our interpersonal and subjective meaning made of perception. Individual differences in the mentalization process along with cognitive and emotional responses to dissociation and PTSD symptoms might be critical for both the construction of new management models as well as the development of novel treatment strategies. However, research in this field remains scarce. Thus, the aim of this study was to analyse the relationship between ACEs, mentalizing and dissociation in adult individuals (see Fig. 1). We hypothesized that participants with low mentalizing capacities may report more dissociation and emotional loading associated with psychological symptoms (anxiety, depression, somatization). Furthermore, we assume that mentalizing may be a mediator of the relationship between ACEs and dissociation in adulthood.

## Materials and methods

### Sample and procedure

This is a secondary analysis of data collected in a single center cross-sectional telephone-based interview study. Study participants for this study were recruited from a larger sample of a previous study^20^. In this previous study, approximately 2600 general hospital patients were screened for experiences of domestic violence, ACEs, and a range of current physical and mental health problems. Among the previous participants who consented to being contacted for future studies, we identified those with experiences of interpersonal violence and randomly drew an age- and sex-matched control sample who had reported neither domestic violence nor ACEs. Further details on study procedures can be found in the previous publication^21^. The study design was in accordance with the Declaration of Helsinki (1964) and its later amendments and was approved by the research ethics committee of the Medical University of Innsbruck (1108/2020).

### Measures

#### Maltreatment and Abuse Chronology of Exposure Scale (MACE)

Adverse childhood experiences (ACEs) were assessed with the German version of the *Maltreatment and Abuse Chronology of Exposure Scale* (MACE)^22^. It consists of 75 items that retrospectively assess the severity of exposure to ten types of maltreatment during each year of childhood and adolescence up to age 18. For each year separately, participants are asked to endorse whether maltreatment occurred during that particular year of their life or not. Thus, onset and cumulative exposure are measured. The MACE provides an overall severity score and multiplicity score (number of types of maltreatment experienced) and has good test–retest reliability and validity^22^. The internal consistency for the MACE total score was excellent (α = 0.92).

#### Essener Trauma Inventory (ETI)

To assess trauma-related symptoms, the symptom list of the Essener Trauma Inventory (ETI) was used. The symptom list of the ETI consists of 23 items rated on a four-point Likert scale. A total score (range: 0 to 69) and four subscales (dissociation; intrusion; avoidance; hyperarousal) can be calculated, with higher values indicating more distress. Values > 16 on the ETI total score indicate a notable distress level and values > 27 a clinically relevant level of PTSD symptoms. Good internal consistency and validity have been reported for the total score and for the four subscales^23^. An excellent internal consistency (α = 0.93) for the ETI-total score was found in our sample.

#### Brief Symptom Inventory (BSI)

Psychological distress was assessed with the Brief Symptom Inventory (BSI-18), consisting of 18 items rated on a four-point Likert scale (from “not at all” to “very often”). A total score and three subscale scores (depression, anxiety, somatization) can be calculated. Good reliability and validity for the subscales and total score have been reported. In our sample, excellent internal consistency was found for the BSI total score (α = 0.92) as well as for the anxiety (α = 0.80) and depression (α = 0.85) subscales.

#### Mentalization Questionnaire (MZQ)

The validated German version of the Mentalization Questionnaire (MZQ) was used to assess the participants self-rated mentalizing^24^. It consists of 15 items rated on a 5-point Likert scale (from “totally disagree” to “totally agree”). A total score can be calculated with higher score indicating robust and lower scores impaired mentalizing^24^. Good internal consistency and validity has been reported for the total score of the MZQ^24^. A good internal consistency for the MZQ total score was observed in our sample (α = 0.84).

### Statistical procedure

Sample characteristics and distribution of mentalization are presented by descriptive statistics. Differences in mentalization, trauma-related symptoms (ETI subscales) between patients with and without ACEs were investigated with independent sample t-tests. Associations of mentalization with dissociation, trauma-related symptoms (ETI subscales), and psychological distress (BSI subscales) were assessed by calculation of Pearson correlation coefficients. Effect sizes of *r* > 0.1 and *d* > 0.2 were considered small, while *r* > 0.3 and *d* > 0.5 indicated a medium, and r > 0.5 and *d* > 0.8 a large effect, respectively.

To investigate if mentalization mediates the association between ACEs and dissociation, the SPSS PROCESS macro tool based on the mediation method with 10,000 bootstrap bias-corrected 95% confidence intervals (CI) was used as recommended by Hayes^25^. In this approach, the indirect effect (i.e., the mediation) is statistically significant if the bootstrapped 95% CI does not include the value zero. Direct and indirect effects are presented as standardized coefficients. R^2^ represents the explained variance of the variable and is presented in percentage values. A priori sample size calculations indicated that a sample of n = 68 patients should be large enough to detect associations of medium effect size (f^2^ = 0.15; α = 0.05; 1 − β = 0.8) in a multiple regression setting with two predictors. Sample size calculations was performed with G*Power (v3.1) and statistical analyses with IBM SPSS (v22.0). P-values < 0.05 (two-sided) were considered statistically significant.

### Statement of ethics

The study was conducted according to the guidelines of the Declaration of Helsinki, and approved by the Ethical Committee of the Medical University Innsbruck (1108/2020).

### Informed consent statement

Informed consent was obtained from all subjects involved in the study.

## Results

A total of 102 participants were contacted between April 14th and 30th 2020, of which 67 (65.7%) agreed to take part in the study. Reasons for non-participation were lack of interest (60.0%), lack of time (22.9%), grave physical or mental health problems (8.6%), language barrier (2.9%), not wanting to conduct a phone interview (2.9%) or immediately hanging up (2.9%) (see Table 1). Participants and non-participants did not statistically differ in regard to previously assessed overall number of ACEs (2.0 vs. 2.7), domestic violence (6.9 vs. 6.3 points), age (48.5 vs. 43.4 years), or sex (female: 65.7% vs. 76.1%) (all p > 0.05).

**Table 1:** Sociodemographic data.

	Mean/n	(SD/%)
Age (years)	48.5	(13.6)
**Gender**		
Male	16	(23.9%)
Female	51	(76.1%)
**Relationship status**		
Single	16	(23.9%)
Married/long-term relationship	40	(59.7%)
Widowed	3	(4.5%)
Divorced/separated	8	(11.9%)
**Living situation**		
Living alone	20	(29.9%)
Living with partner/family	42	(62.7%)
Living with family of origin	4	(6.0%)
Living in shared apartment	1	(1.5%)
**Parenthood**	44	(65.7%)
Missing data	1	(1.5%)
**Social class**		
Lower class/working class	12	(17.9%)
Middle class	45	(67.2%)
Upper class	10	(14.9%)

Mean age of the participants was 48.5 years, the majority was female (76.1%), living with their partner or family (62.7%) and about two thirds of the sample had children.

### Association of mentalization with ACEs, dissociation, PTSD symptoms and psychological distress

Among the included patients, n = 37 patients (55.2%) reported no ACEs, while the remaining n = 30 (43.3%) reported one or more forms of ACEs. Mean ACE score was 2.0 (SD = 2.5) and patients reported a mean MZQ total score of 3.8 points (SD = 0.7). As for dissociation, a mean score of 3.0 (SD = 3.6) was found in the sample. Patients with ACEs showed a significantly decreased ability of mentalization with a large effect size (4.1 vs. 3.4 points; t = 4.2; p < 0.001; d = 1.05) and higher dissociation scores with a medium effect size (4.4 vs. 1.8 points; t = 3.0; p = 0.005; d = 0.75).

Mentalization and dissociation were highly significant correlated with a large effect size (r = − 0.70, p < 0.001). Additionally, significant associations with large effect sizes could be observed between mentalization and all three PTSD-subscales (intrusion: r = − 0.53; avoidance: r = − 0.70; hyperarousal: r = − 0.60; all p < 0.001) as well as with depression (r = − 0.64; p < 0.001) and anxiety (r = − 0.64; P < 0.001). The association of mentalization and somatization was also statistically significant with a medium effect size (r = − 0.46, p < 0.001).

### Mentalization as mediator between ACEs and dissociation

**Figure 2:**
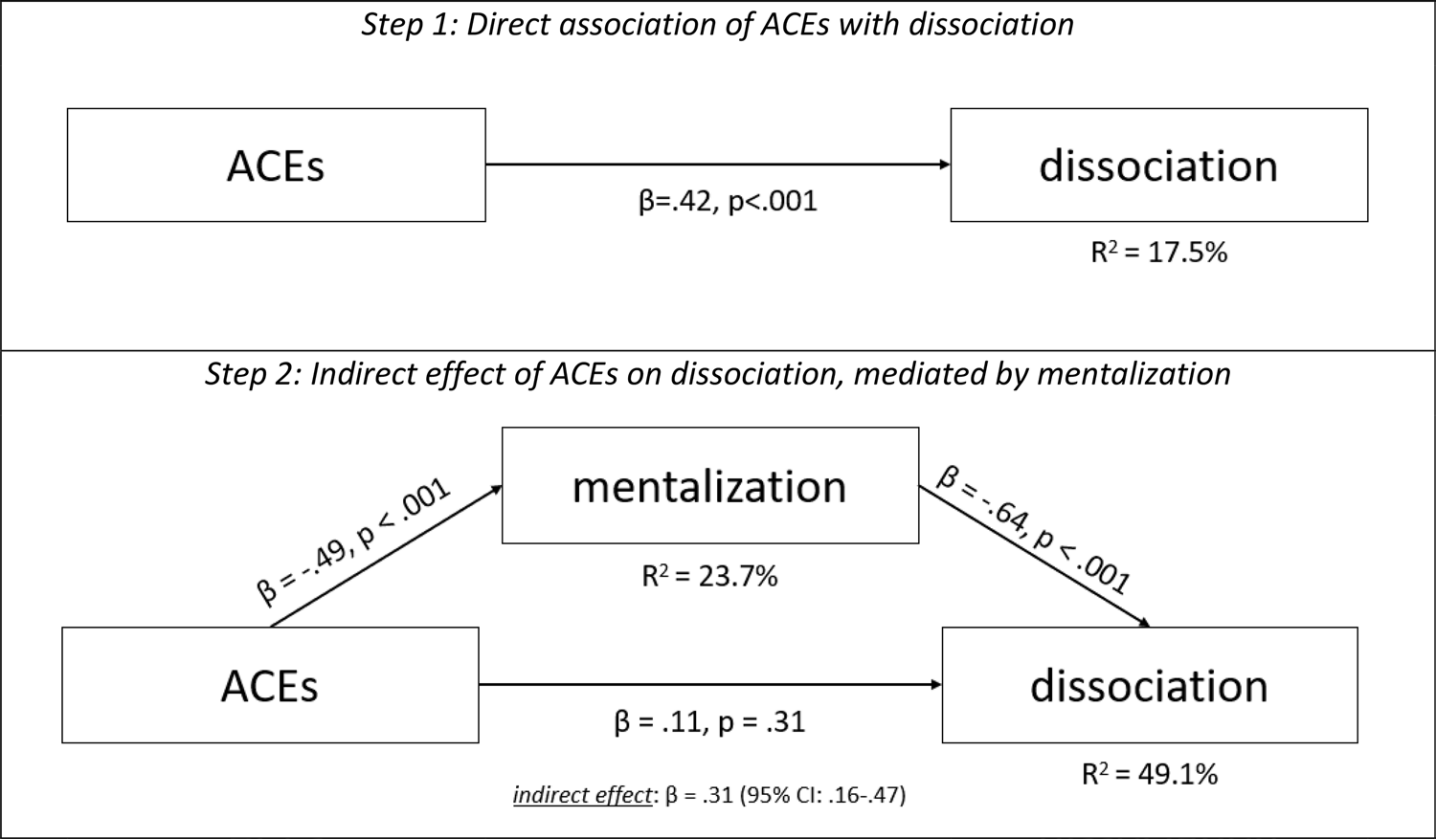
Mediation analysis of the direct and indirect association of ACEs with dissociation, mediated by mentalization. ACEs = Adverse childhood experiences, β = standardized coefficient; R2 = explained variance of the variable.

To test the association of ACEs, mentalization and dissociation, a mediation analysis was conducted. In a first step, the direct association of ACEs on dissociation was tested. ACEs were significantly associated with dissociation (β = 0.42, p < 0.001) and predicted 17.5% of its variance. In a second step, mentalization was added to the model as mediator between ACEs and dissociation. Higher ACE values were associated with lower mentalization scores (β = − 0.49, p < 0.001) and predicted 23.7% of its variance. While mentalization was significantly associated with dissociation (β = − 0.64, p < 0.001), the association of ACEs with dissociation was no longer significant (β = 0.11, p = 0.31) and the indirect effect was statistically significant (β = 0.32, 95% CI 0.16–0.47). The explained variance of dissociation notably increased to 49.1% when mentalization was included as mediator in the model. Thus, the data indicated that the association of ACEs on dissociation was fully mediated by mentalization. See also Fig. 2.

## Discussion

The aim of our study was to assess the relationship between ACEs, mentalizing and dissociation in adult individuals. In our sample, there was a clear association of ACEs with dissociative symptoms. However, the direct association of ACEs on dissociation was not longer statistically significant when mentalization was added to the model as a mediator of said relationship.

In accordance with previous literature, we found a significant association between dissociative symptoms and mentalization, which can be understood as a dysfunctional adaptation process to a traumatic environment^26^ with disruption of biobehavioral mechanism related to self and identity. Dysfunctional and maladaptive coping strategies influencing the stress response with so called stress depending changes from explicit to an implicit mentalizing process^27^. A stress dependent switching from explicit to implicit mentalizing comes to bear when coping strategies are no longer sufficient to regulate personal stress arousal in the interaction with the attachment system.

From the perspective of mentalization theory, the typical emotion regulation in patients with a borderline personality disorder is seen as an imperfect balance between the cognitive and affective poles as well as the explicit and implicit mentalization poles. An individuals’ on the effects of dysfunctional behavior on self and others is limited. In the case of interpersonal stress, it would be more functional if controlled and cognitive mentalizing (explicit) were available to the individuals. According to psychodynamic theory, a patient with a lower personality functioning based on maladaptive early attachment experiences shows symptom intensification and a lower therapy response^28^. A broad range of research show that ACEs can be considered as a transdiagnostic factor with implications in a variety of emotional and functional disorders and problems^29^. The impact of traumatic experience plays a key role in the regulation of distress based on the attachment system^30^ with subsequent problems in mentalizing. Social embedded stress response may enforce the pathway between dissociation and mentalization in patients with attachment insecurity. This theory is supported by a recent study, which showed that attachment insecurity in combination with lower mentalizing mediated the link between childhood trauma and PTSD symptoms in adults who had experienced childhood neglect and abuse^31^. Early maltreatment may increase the risk for the development of attachment insecurity with long-lasting effects. The development of mentalizing is thought to be fostered by secure attachment relationship with a process of contingent mirroring of child’s affects and subjective emotional experiences. This mental representation is important for strategies to regulate stress and to communicate with the self and the other^32^. Sharp et al. describe a social-cognitive model of PTSD and attachment insecurity with impaired mentalizing abilities mediating associations with dissociative experiences^33^. Dissociative mental representations engendered by childhood trauma could be understood as as a dysfunctional adaptive function to minimize the fear of shame and guilty. The mentalizing ability could lead to a damping of this social-cognitive model of PTSD and dissociative experiences. Social embedded mentalizing may influence important aspects of individual function.

Our data also demonstrate that lower mentalization was associated with higher depression, anxiety and somatization as well as PTSD symptoms (avoidance, hyperarousal, intrusions). Depression and depressive symptoms with impairments in interpersonally transmitted information may be highly linked to mentalizing abilities based on developmental aspects of attachment and social relationships. A few empirical studies reported associations linking change in interpersonal functioning and attachment security with change in depressive symptoms^34^. Relevant early developmental epigenetic modification of gene expression influences behavioral and emotional patterns in patients with depressive symptoms^35^. This biobehavioral mechanism involved in problems related to self and identity should be a part of social capacity treatments. Interpersonal therapy focusing on helping patients to create or renew social contacts and social support may benefit from better mentalizing^36^. Mentalization with its linkages to attachment theory offers possibilities for understanding the dynamics of depression regarding differences in severity and course of disease^37^. Functional domains, that are often impaired in patients with ACEs, include personality functioning, affective, cognitive and self-regulatory resources as well as the quality of the self-other representation^36^. These functional domains are important for the dynamic interplay of meaningful relationships.

Somatization refers to psychological stress caused by the perception of physical dysfunctions focusing on body symptoms with strong autonomous mediation^38^. Subjective perception, thoughts, emotions and behavior associated with the individual somatic status are sometimes clinically more important than a medical diagnosis^39^. The specific weight for the patients' perception is a body-related cognitive ability or competence, justified from a neuroscientific and a health science perspective. Interoceptive awareness and conscious body-related self -regulation seems to be a basic function, which may serve in patients with ACEs as an important homeo-static/allostatic control^40^. Subjective representations of illness determine the coping behaviors and consequently the illness outcome. Somatization thus may be viewed as a primary driver for higher perception, symptom reporting, health care use, symptom persistence, and negative treatment outcome^41^.

Internalized traumatic early experiences (as often the case when experiencing ACEs) may lead to corresponding working models and obstructions in the functional regulation of emotions with less flexibility and more adaptive personality patterns^42^. Overcontrol of emotions such as in the dissociative subtype of PTSD need effective treatments as fostering mentalizing with a top-down regulation^43^ and strengthening the individuals capacity to feel and to simultaneously reflect on his or her feelings.

Impaired mentalization in individuals with posttraumatic symptoms is associated with more psychological distress and higher symptom perception^43^. Similarly, individuals with ACEs and an insecure attachment style show more psychological stress and higher symptom load. The modulation of primary affective states into cognitive -affective key features is disrupted by attachment trauma with leading to impairments in the capacity for self-regulation and the capacity for accessing the adaptive functioning of the social imaginations in relation to the intersubjective self^12^. Understanding my inner world and the world of the other with mentalizing capacities can contribute to the understanding of psychological resilience.

We are essentially tied to who or what we consider ourselves to be. The images we make of our selves characterize who or what we are^44^.

The present study has several strengths and limitations. A major limitation is the limited sample size. Even though the sample size calculations indicated that the sample was sufficiently powered to detect medium effect sizes and results indicated positive findings, results should be interpreted with caution with regard to a possibly reduced variability of the scales’ scores. In addition, we did not assess attachment (insecurity) in our study. Given that previous research has shown the close association between ACEs, attachment and mentalization, attachment might be an important additional component in a mediation model addressing adult psychopathology. Future research should address the role of attachment in the interplay of childhood trauma, adult dissociation and mentalization as a mediator. To our knowledge, this is the first study to investigate the mediation effect of mentalization in the association between ACEs and dissociation, which is a major strength of this study. A second strength is the applied study methodology: in the present study carefully telephone-based structured interviews were conducted by highly trained specialists in psycho-traumatology. This approach was not only chosen to improve the quality of collected data, but also to guarantee safety of this highly vulnerable patient collective and to support affected persons if necessary.

## Conclusion

The relationship between ACEs and dissociation is fully mediated by mentalization; this means than the important predictive factor for dissociation is not if you have been abused as a child, but rather how this abuse or neglect influenced your mentalization capabilities. We suggest that mentalizing is a helpful transtheoretical and transdiagnostical concept to explain vulnerability to dissociation and its treatment.

Additionally, this indicates that early treatment of individuals affected by ACEs with a focus to foster the development of mentalization could prevent from developing dissociative symptoms as an adult.

Social embedded treatment strategies with adaptive functioning of social imaginations in relation to inter and intrasubjective capabilities may foster a therapeutic outcome.

## References

[1] Schimmenti A (2018). The trauma factor: Examining the relationships among different types of trauma, dissociation, and psychopathology. J. Trauma Dissoc..

[2] Heim C, Nemeroff CB (2001). The role of childhood trauma in the neurobiology of mood and anxiety disorders: Preclinical and clinical studies. Biol. Psychiatry.

[3] Tottenham N, Sheridan MA (2010). A review of adversity, the amygdala and the hippocampus: A consideration of developmental timing. Front. Hum. Neurosci..

[4] Vonderlin R, Kleindienst N, Alpers GW, Bohus M, Lyssenko L, Schmahl C (2018). Dissociation in victims of childhood abuse or neglect: A meta-analytic review. Psychol. Med..

[5] Ruf M, Schauer M, Neuner F, Catani C, Schauer E, Elbert T (2010). Narrative exposure therapy for 7- to 16-year-olds: A randomized controlled trial with traumatized refugee children. J. Trauma Stress.

[6] McGowan PO, Sasaki A, D'Alessio AC, Dymov S, Labonte B, Szyf M (2009). Epigenetic regulation of the glucocorticoid receptor in human brain associates with childhood abuse. Nat. Neurosci..

[7] Fonagy P, Luyten P, Allison E, Campbell C (2017). What we have changed our minds about: Part 2. Borderline personality disorder, epistemic trust and the developmental significance of social communication. Borderline Personal. Disord. Emot. Dysregul..

[8] Taubner S, Volkert J (2016). Mentalisierungsbasierte Therapie für Adoleszente (MBT-A).

[9] Apperly IA, Butterfill SA (2009). Do humans have two systems to track beliefs and belief-like states?. Psychol. Rev..

[10] Mikulincer M, Shaver PR (2012). An attachment perspective on psychopathology. World Psychiatry.

[11] Mikulincer M, Shaver PR, Solomon Z (2015). An attachment perspective on traumatic and posttraumatic reactions. Future Directions in Post-traumatic Stress Disorder.

[12] Fonagy P, Luyten P, Allison E, Campbell C (2019). Mentalizing, epistemic trust and the phenomenology of psychotherapy. Psychopathology.

[13] Lupien SJ, McEwen BS, Gunnar MR, Heim C (2009). Effects of stress throughout the lifespan on the brain, behaviour and cognition. Nat. Rev. Neurosci..

[14] Williams LM, Liddell BJ, Kemp AH, Bryant RA, Meares RA, Peduto AS, Gordon E (2006). Amygdala–prefrontal dissociation of subliminal and supraliminal fear. Hum. Brain Mapp..

[15] Lanius RA, Vermetten E, Loewenstein RJ, Brand B, Schmahl C, Bremner JD, Spiegel D (2010). Emotion modulation in PTSD: Clinical and neurobiological evidence for a dissociative subtype. Am. J. Psychiatry.

[16] Teicher MH, Samson JA, Anderson CM, Ohashi K (2016). The effects of childhood maltreatment on brain structure, function and connectivity. Nat. Rev. Neurosci..

[17] Fonagy P, Bateman A (2008). The development of borderline personality disorder—A mentalizing model. J. Pers. Disord..

[18] Panksepp J, Solms M (2012). What is neuropsychoanalysis? Clinically relevant studies of the minded brain. Trends Cogn. Sci..

[19] Hopwood CJ, Zimmermann J, Pincus AL, Krueger RF (2015). Connecting personality structure and dynamics: Towards a more evidence-based and clinically useful diagnostic scheme. J. Pers. Disord..

[20] Riedl D, Exenberger S, Daniels JK, Bottcher B, Beck T, Dejaco D (2019). Domestic violence victims in a hospital setting: Prevalence, health impact and patients' preferences—results from a cross-sectional study. Eur. J. Psychotraumatol..

[21] Riedl D, Beck T, Exenberger S, Daniels J, Dejaco D, Unterberger I (2019). Violence from childhood to adulthood: The influence of child victimization and domestic violence on physical health in later life. J. Psychosom. Res..

[22] Teicher MH, Parigger A (2015). The 'Maltreatment and Abuse Chronology of Exposure' (MACE) scale for the retrospective assessment of abuse and neglect during development. PLoS ONE.

[23] Tagay S, Dullmann S, Hermans E, Repic N, Hiller R, Senf W (2011). The Essen Trauma-Inventory for children and adolescents (ETI-CA). Z Kinder Jugendpsychiatr. Psychother..

[24] Hausberg MC, Schulz H, Piegler T, Happach CG, Klopper M, Brutt AL (2012). Is a self-rated instrument appropriate to assess mentalization in patients with mental disorders? Development and first validation of the mentalization questionnaire (MZQ). Psychother. Res..

[25] Hayes AF (2018). Introduction to Mediation, Mederation and Conditional Process Analysis: A Regression-Based Approach.

[26] Luyten P, Campbell C, Allison E, Fonagy P (2020). The mentalizing approach to psychopathology: State of the art and future directions. Annu. Rev. Clin. Psychol..

[27] Taubner S (2020). Parental mentalizing as a key resource for difficult transitions. Attach. Hum. Dev..

[28] Zimmermann J, Dahlbender RW, Herbold W, Krasnow K, Turrion CM, Zika M (2015). The OPD structure questionnaire captures the general features of personality disorder. Psychother. Psychosom. Med. Psychol..

[29] Luyten P, Vliegen N, Van Houdenhove B, Blatt SJ (2008). Equifinality, multifinality, and the rediscovery of the importance of early experiences: Pathways from early adversity to psychiatric and (functional) somatic disorders. Psychoanal. Study Child.

[30] Bateman A, Campbell C, Luyten P, Fonagy P (2018). A mentalization-based approach to common factors in the treatment of borderline personality disorder. Curr. Opin. Psychol..

[31] Huang YL, Fonagy P, Feigenbaum J, Montague PR, Nolte T, London Personality and Mood Disorder Research Consortium (2020). multidirectional pathways between attachment, mentalizing, and posttraumatic stress symptomatology in the context of childhood trauma. Psychopathology.

[32] Shipman K, Zeman J, Penza S, Champion K (2000). Emotion management skills in sexually maltreated and nonmaltreated girls: A developmental psychopathology perspective. Dev. Psychopathol..

[33] Sharp C, Penner F, Ensink K (2020). Reflective function and borderline traits in adolescents. J. Pers. Disord..

[34] Markowitz JC, Skodol AE, Bleiberg K (2006). Interpersonal psychotherapy for borderline personality disorder: Possible mechanisms of change. J. Clin. Psychol..

[35] Jiang S, Postovit L, Cattaneo A, Binder EB, Aitchison KJ (2019). Epigenetic modifications in stress response genes associated with childhood trauma. Front. Psychiatry.

[36] Markowitz JC, Milrod B, Luyten P, Holmqvist R (2019). Mentalizing in interpersonal psychotherapy. Am. J. Psychother..

[37] Fischer-Kern M, Tmej A (2019). Mentalization and depression: Theoretical concepts, treatment approaches and empirical studies—An overview. Z. Psychosom. Med. Psychother..

[38] Franke GH, Jaeger S, Glaesmer H, Barkmann C, Petrowski K, Braehler E (2017). Psychometric analysis of the brief symptom inventory 18 (BSI-18) in a representative German sample. BMC Med. Res. Methodol..

[39] Carrozzino D, Bech P, Patierno C, Onofrj M, Morberg BM, Thomas A (2017). Somatization in Parkinson's disease: A systematic review. Prog. Neuropsychopharmacol. Biol. Psychiatry.

[40] Herpertz SC, Fuchs T (2014). Interactions between neuroscience and psychopathology. Psychopathology.

[41] Barrett LF, Simmons WK (2015). Interoceptive predictions in the brain. Nat. Rev. Neurosci..

[42] Spinhoven P, Elzinga BM, Van Hemert AM, de Rooij M, Penninx BW (2016). Childhood maltreatment, maladaptive personality types and level and course of psychological distress: A six-year longitudinal study. J. Affect. Disord..

[43] Kim S, Fonagy P, Allen J, Strathearn L (2014). Mothers’ unresolved trauma blunts amygdala response to infant distress. Soc. Neurosci..

[44] Gabriel M (2017). I am Not a Brain: Philosophy of Mind for the 21st Century.

